# An unique dataset for Christian sacral objects identification

**DOI:** 10.1016/j.dib.2023.109137

**Published:** 2023-04-11

**Authors:** Marie Feslová, Michal Konopa, Kateřina Horníčková, Jiří Jelínek, Eliška Píšová, Radka Bunešová, Jan Fesl

**Affiliations:** a University of South Bohemia, Faculty of Science, Department of Informatics, Branišovská 31a, 370 05, Czech Republic; b University of South Bohemia, Faculty of Arts, Institute of Arts and Culture Studies, Branišovská 31a, 370 05, Czech Republic

**Keywords:** Christian sacral objects, Machine learning, Image processing, Identification, Middle age

## Abstract

Christian religious monuments as cathedrals, chapels, and temples, are found in many places on our planet. World-famous buildings such as the Notre Dame Cathedral in Paris, Gaudi's Cathedral in Barcelona, and St. Vitus Cathedral in Prague are commonly known. Many online photographs can be used to build machine-learning models to identify them. The number of photographs is already significantly lower for little-known buildings, such as small churches in the Czech-German border region, and similar approaches cannot be used for identification. Based on these facts, our team has compiled a unique dataset for identifying the most important elements of Christian sacral buildings as altars, frescoes, pulpits, etc., which are almost always found in them. Our data set was manually created from several thousand real photographs. This dataset seems to be very usable, e.g., for creating new machine learning models and identifying objects in sacred objects or the objects themselves.

**Specifications Table**

**Table:** 

Subject:	Humanities (General)
Specific subject area:	Christian Middle-Age Churches and Their Architecture
Type of data:	Image (PNG files) Textual (CSV files using ';' as a separator)
How the data were acquired:	The original images were generated by 6 photographers (members of our team) using similar mobile camera types. The image data was then manually preprocessed and filtered, resp. assigned to a specific category. The in-house developed tool "PhotoCutter" was used for image preprocessing and classification*.*
Data format:	Raw Textual
Description of data collection:	First, the interiors of the selected churches were photographed. From these photographs, using the PhotoCutter software tool, the cut-outs of sacral objects of the proposed categories were created and exported to PNG format. The summary CSV file contains a comprehensive list of images and their parameters.
Data source location:	Department of Informatics, Faculty of Science, University of South Bohemia. Branišovská 31a, České Budějovice, Czech Republic, 37,001. GPS Coordinates: 48° 58′ 28.09″ N, 14° 28′ 27.62″ E
Data accessibility:	Repository name: Zenodo Data identification number: 10.5281/zenodo.7653605 Direct URL to data: https://zenodo.org/record/7653605

## Value of the Data

• To the best of our knowledge, the dataset is the one of the first containing a significant amount of samples of the various object categories related to Christian sacral objects. Another similar dataset exists [1], including a lower count of object categories and image samples with lower resolution, color depth and size. The motivation and research interest related to the dataset [1] can be found in [2]. Compared to [1], our dataset targets more the interior specific unique identifiable objects in Christian sacral buildings such as frescos, organs, baptismal fonts, etc. Our dataset substantially extends the essential object category list introduced in [1] suitable for unambiguous identification of sacral buildings.
• Researchers and application developers can utilize the data to create machine-learning models to classify individual types of sacral objects.
• Anyone working on identifying and classifying sacral objects can append this set to their data and refine the classification of the objects. Furthermore, he can also focus on identifying and classifying sacral objects of categories not listed in this set.
• The dataset could be usable for the study, analysis, signs identification in the area of architecture, building conservation and history.

## 1 Objective

Our work is part of a larger cross-border cooperation project that aims at developing an application [3] providing local information on cultural heritage monuments in the area. The project aims to support the use of mobile technologies in the field of tourism and cultural heritage. From the computer science point of view, the application seeks to identify a specific sacral object that is wholly or partially depicted in the input image, ideally with success comparable to human capacity. Identifying a specific object that is only partially depicted in digital photography is a non-trivial task currently being solved by several different commercial services. These services work for unidentified photographs depicting different types of objects (e.g., people, animals, buildings, etc.) with very different levels of reliability. Unfortunately, to the best of our knowledge, there is no service today that can identify individual sacral objects with sufficient accuracy. For this reason, our team has created a unique dataset and designed a multi-step identification scheme using advanced machine learning methods.

## 2 Data Description

We selected some essential details and building elements for the test set based on our professional experience, e.g. statue, fresco, altar, pulpit, tower, window, etc. It is also essential to combine them - only their combination forms a unique identification of a given building and the basis for the building's temporal, stylistic, and formal classification and evaluation, as well as for the actual identification of the building. Because of their form, wall paintings are easily identifiable, which is why contemporary church paintings were also included among the elements. The church can be identified according to the stylistic level and iconography of the paintings in the interior.

The complete list of categories can be found in Table 1. The graphs in the right column represent the image distribution histogram ordered by the image area expressed in kilopixels (Kpix).

**Table 1:** The overview of all image categories contained within the data set with a practical category visualization.

The entire content of the data set is placed within one directory. The file list contains two file types - CSV and PNG. There is also the sub-folder “Original” that contains the original source image files used for creation of category samples (Table 2).

Each contained PNG file name has the following name structure: {X_Y.PNG}, where X is the number identifier of a specific category and Y is the image number within the specific category. The summary CSV file contains the structure described in Table 3.

**Table 2:** File types present in the data set.

File Extension	File content
CSV	General information for all files contained within the data set.
PNG	Specific image data stored in PNG format.

**Table 3:** The structure of records contained within the CSV specification file.

Attribute	Description
File Name	The name of the PNG image file.
Category	The number identifier (ID) of the image category.
Additional Info	The textual description of the image category.
Image Width	The PNG image file width in pixels.
Image Height	The PNG image file height in pixels.
Original Image	The name of the source image file from which the file was created. The source image file is located in the sub-folder “Original”.

**Fig. 1:**
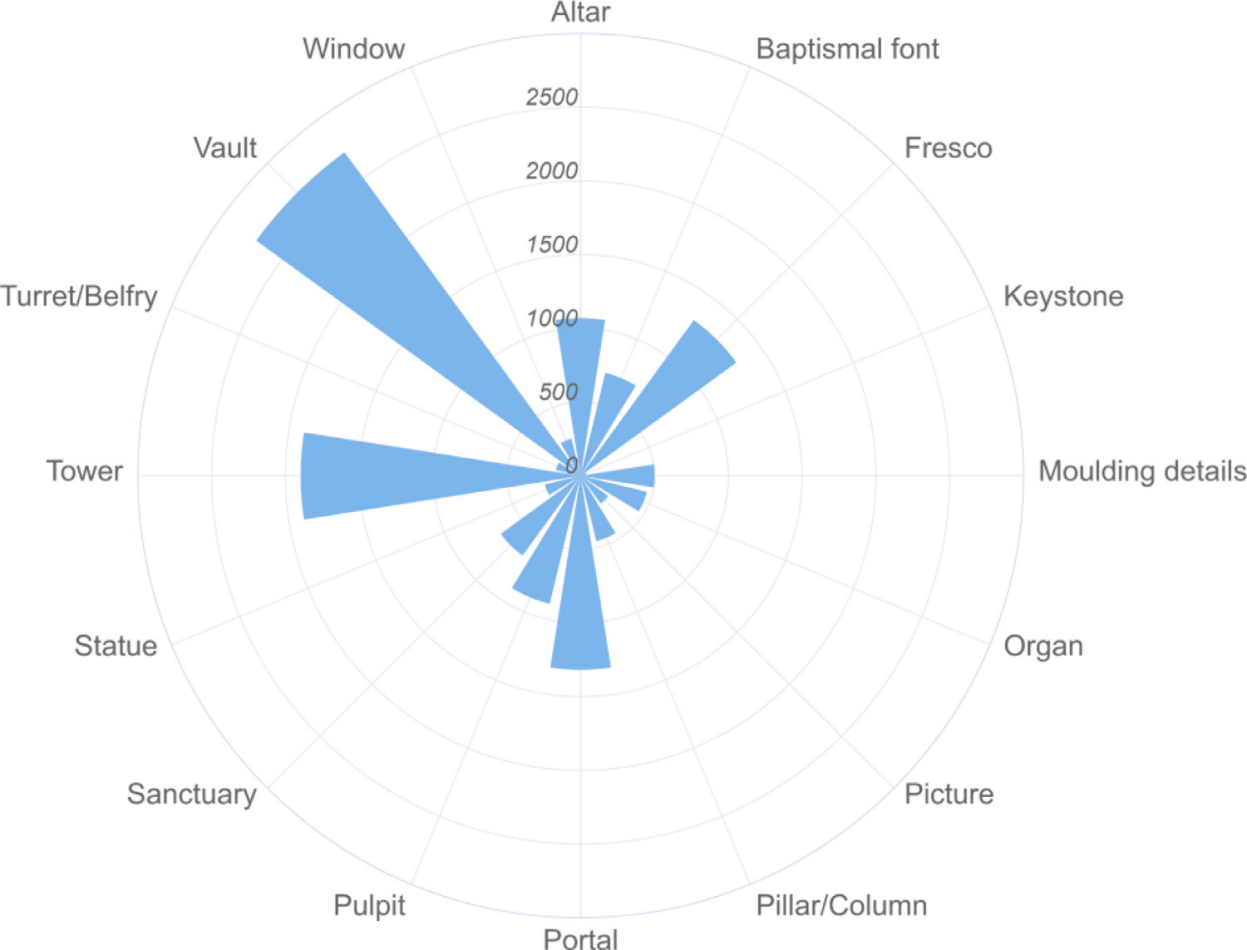
Image data set distribution according to average area size [Kpix] of samples in each category.

**Table 4:** The list of Python scripts used for the final data set creation.

No.	Name (*.py)	Description
1	datasetMerger	The script used for the data set creation (files merging).
2	main-datasetmerger	Initial executable script file.
3	csvMerger	Script for CSV file generation containing the images information.
4	fileSearcher	Script for searching of files to be assigned into the dataset.

**Fig. 2:**
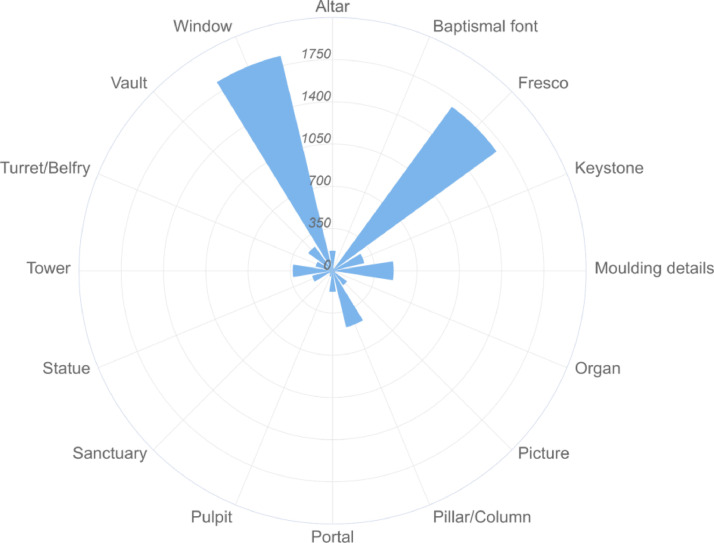
Image data set distribution according to the count of samples in each category.

**Fig. 3:**
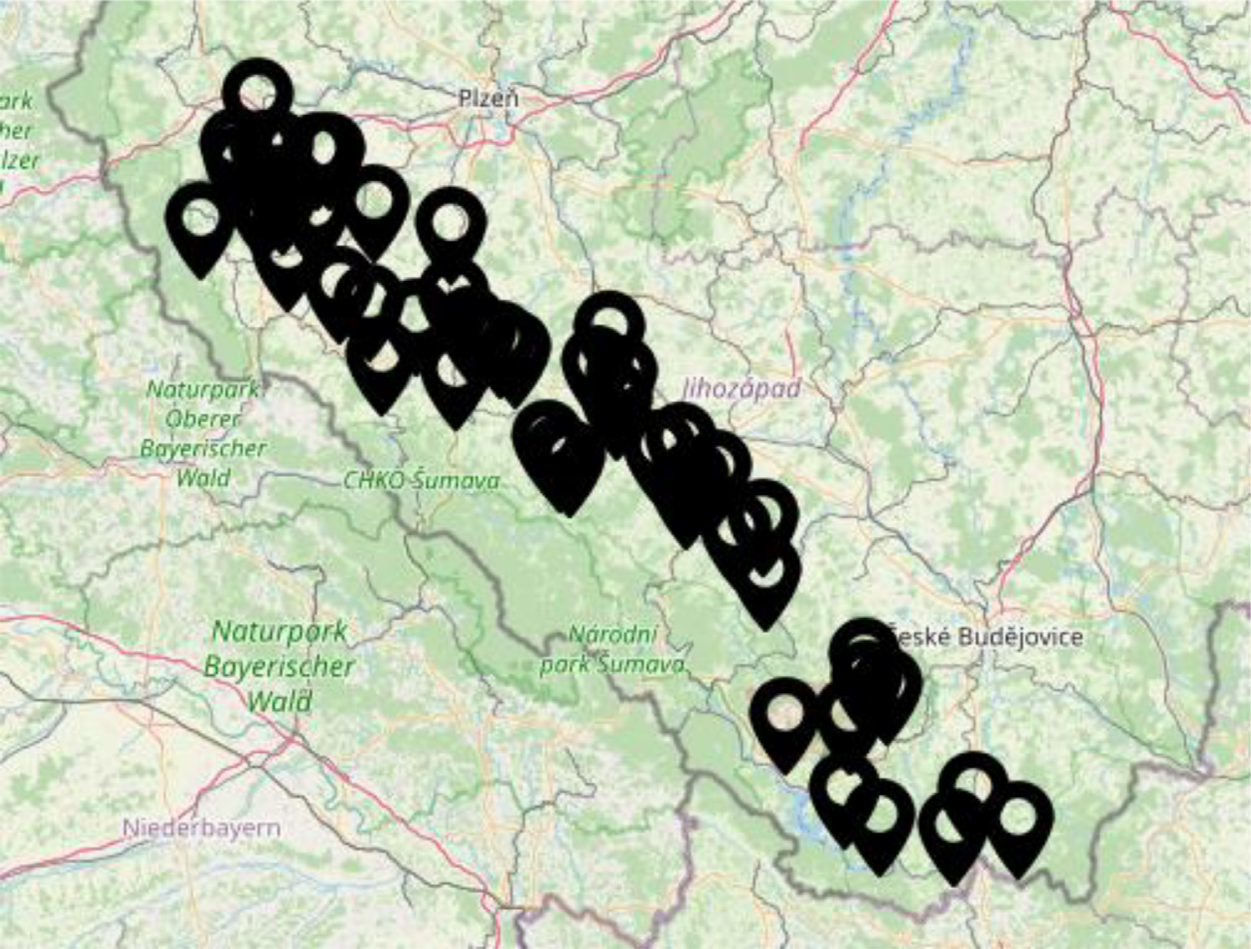
Representation of the location of the churches on the Czech-Bavarian border, which were selected to create the photographs in the dataset.

## 3 Experimental Design, Materials and Methods

The alternation of artistic styles characterizes the development of European art. A style is a coherent artistic expression of an epoch characterized by a coherent combination of specific forms and features typical of a given period, which are applied in work by contemporary artists [4,5]. Style or style constitutes a disjunctive and externally recognizable set of visual signs in which an object is made and forms the formal language of a work, much like graphemes in the written text [5]. We examine these through formal analysis. Some of these elements are easily recognizable, while others are harder to identify because they consist of multiple specific elements' simultaneous occurrence and interplay [[6], [7]].

In architecture, standard features are manifested both in the period's conventional approach to shaping space and in the formal details of the building that identify it. The collection of these elements helps to reveal the structural development of a given building throughout history. The expert's observation and determination are based on the similarity and identification of these characteristic elements and their combinations [8], which are gradually learned through observation during education.

Churches in the Bohemian environment have undergone complex architectural development and many modifications. The resulting structure is often a mixture of details from different eras; medieval (Romanesque and Gothic) forming the basis of the buildings studied in the South Bohemian region, the characteristic clustered Romanesque windows, elongated Gothic windows, Gothic polygonal finial, fractured Gothic triumphal arch or sanctuary, sanctuary tower, square tower, etc.

There was no mass production during the Middle Ages and the early modern period. The building factories created elements unique to the site, corresponding to the morphology of the style of the time, but with the input of the stonemason's inventiveness [9]. Although each detail of the building is individual, since it was created as a unique piece, it bears general characteristics in a form typical of Central European architecture and the region. Thus, it allows us to identify and classify the element to an epoch and a geographical region or a range of buildings, but also to that particular building by its unique features and arrangement (Wölfflin, 1915, 149–150) [4].

At first, we had to identify the suitable churches from the point of view of different attributes like building style, current state, existence of the specific classification features contained in Table 1, etc. The map containing the selected churches can be seen in Fig. 3.

The images contained in our dataset have been collected manually over many months through manual shooting with a digital camera. At first, the experts checked all image data from the point of view of the quality, lighting, resolution, sharpness, etc., and then unsuitable samples were eliminated.

All suitable data were processed by our in-house application called **PhotoCutter**. From each photo, the specific objects were manually selected and assigned to the selected classification categories (see above). The selected objects were exported to the standalone image files into the PNG format into the common structured directory.

Finally, the Python scripts were then run over the created image files to assemble the final dataset and to build the summary CSV file. The There are 4 Python scripts used for the data set processing. The more detailed information can be found in Table 4.

All relevant image files were automatically found within the common structured directory (by imageSearcher), extracted the specific technical image information (like resolution, DPI, compression algorithms, etc.), and created one homogeneous data set directory with many images (by datasetMerger)and generated the CSV meta file (by csvMerger). Our dataset currently contains 6420 images that are classified into 16 categories meant in Table 1. The specific image distribution is described in Figs. 1 and 2.

## Ethics Statements

All images contained in our dataset have been created by the team members and do not come from third-party sources.

Our data does not come from involved human subjects or animal experiments and is not retrieved from social media platforms.

## Credit Author Statement

**Marie Feslová:** Data Curation, Validation, Writing. **Michal Konopa:** Software, Writing. **Kateřina Horníčková:** Formal Analysis, Supervision, Writing. **Jiří Jelínek:** Methodology. **Eliška Píšová:** Data Curation. **Radka Bunešová**: Data Curation. **Jan Fesl:** Supervision, Conceptualization, Methodology, Writing.

## Declaration of Competing Interest

The authors declare that they have no known competing financial interests or personal relationships that could have appeared to influence the work reported in this paper.

## Data Availability

An Unique Dataset for Christian Sacral Objects Identification (Original data) (Zenodo).

## Appendix Supplementary materials

Image, application 1

Image, application 2

Image, application 3

Image, application 4
